# Spontaneous apoptosis in ovarian carcinomas: a positive association with *p53* gene mutation is dependent on growth fraction

**DOI:** 10.1054/bjoc.1999.0967

**Published:** 2000-01-18

**Authors:** J Kupryjánczyk, A Dansonka-Mieszkowska, T Szymánska, G Karpínska, A Rembiszewska, M Rusin, R Konopínski, E Kraszewska, A Timorek, D W Yandell, J Stelmachów

**Affiliations:** Departments of Molecular Biology and Biostatistics, The Maria Skl^′^odowska-Curie Memorial Cancer Center and Institute of Oncology, Roentgena 5, Warsaw, 02-781, Poland; Department of Tumour Biology, Wybrzez.e Armii Krajowej 15, Gliwice, 4-100, Poland; Departments of Pathology and Obstetrics and Gynecology, Medical Academy Warsaw, Bródnowski Hospital, Kondratowicza 8, Warsaw, 03-242, Poland; University of Vermont College of Medicine, Pathology Department and Vermont Cancer Center, Burlington, VT, 05405, USA

**Keywords:** apoptosis, *p53* gene mutation, p53 protein accumulation, growth fraction, bcl-2, ovarian cancer, survival

## Abstract

Changes in cell survival contribute to tumour development, influence tumour biology and its response to chemotherapy. *p53* gene alterations should negatively affect apoptosis by impaired p53-dependent apoptotic response. We looked for associations between spontaneous apoptosis, *p53* gene mutation, p53 protein accumulation, growth fraction, bcl-2 expression and histological parameters in 64 ovarian, four tubal and three peritoneal carcinomas. Apoptotic cells were detected with the TUNEL method. *p53* gene variants were detected by the single-strand conformation polymorphism and were sequenced directly. P53, Ki-67 and bcl-2 protein expressions were detected immunohistochemically. A weighed multiple logistic regression model was applied. Apoptotic index (Al) ranged 0.02–0.18 (mean 0.11); proliferation index (PI) ranged 3–90% (mean 54%). *p53* gene mutations were present in 51, p53 protein accumulation in 46, and diffuse bcl-2 expression in 29 of 71 tumours. The AI was positively associated with the presence of *p53* gene mutation (*P* = 0.011). However, the PI included into the analysis did positively influence the AI (*P* = 0.02) and diminished the association with *p53* gene mutation (*P* = 0.082). The AI was negatively associated with good histological differentiation (*P* = 0.0006), the serous tumour type (*P* = 0.002), and diffuse bcl-2 expression (*P* = 0.025). Strong bcl-2 expression was associated with endometrioid tumour type (*P* = 0.002). FIGO stage and p53 protein accumulation were the only parameters that influenced overall survival time. Thus, our results suggest that histological tumour type and grade are major determinants of spontaneous apoptosis in ovarian carcinomas;*p53* alterations do not adversely but rather positively affect spontaneous apoptosis by increasing growth fraction. This, in turn, suggests p53-independency of spontaneous apoptosis in ovarian carcinomas. © 2000 Cancer Research Campaign


British Journal of Cancer (2000) **82**(3), 579–583
© 2000 Cancer Research Campaign
DOI: 10.1054/ bjoc.1999.0967, available online at http://www.idealibrary.com on IDEAL®

### Spontaneous apoptosis in ovarian carcinomas: a positive association with *p53* gene mutation is dependent on growth fraction

**J Kupryjańczyk[1,4], A Dansonka-Mieszkowska[1], T Szymańska[1], G Karpińska[4], A Rembiszewska[1], M Rusin[3], R Konopiński[1], E Kraszewska[2], A Timorek[5], DW Yandell[6] and J Stelmachów[5]**

Departments of [1]Molecular Biology and [2]Biostatistics, The Maria Skłodowska-Curie Memorial Cancer Center and Institute of Oncology, Roentgena 5, 02-781 Warsaw, Poland; [3]Department of Tumour Biology, Wybrzeże Armii Krajowej 15, 4-100 Gliwice, Poland; Departments of [4]Pathology and [5]Obstetrics and Gynecology, Medical Academy Warsaw, Bródnowski Hospital, Kondratowicza 8, 03-242 Warsaw, Poland; [6]University of Vermont College of Medicine, Pathology Department and Vermont Cancer Center, Burlington, VT 05405, USA

**Summary** Changes in cell survival contribute to tumour development, influence tumour biology and its response to chemotherapy. *p53* gene alterations should negatively affect apoptosis by impaired p53-dependent apoptotic response. We looked for associations between spontaneous apoptosis, *p53* gene mutation, p53 protein accumulation, growth fraction, bcl-2 expression and histological parameters in 64 ovarian, four tubal and three peritoneal carcinomas. Apoptotic cells were detected with the TUNEL method. *p53* gene variants were detected by the single-strand conformation polymorphism and were sequenced directly. P53, Ki-67 and bcl-2 protein expressions were detected immunohistochemically. A weighed multiple logistic regression model was applied. Apoptotic index (AI) ranged 0.02–0.18 (mean 0.11); proliferation index (PI) ranged 3–90% (mean 54%). *p53* gene mutations were present in 51, p53 protein accumulation in 46, and diffuse bcl-2 expression in 29 of 71 tumours. The AI was positively associated with the presence of *p53* gene mutation ($P = 0.011$). However, the PI included into the analysis did positively influence the AI ($P = 0.02$) and diminished the association with *p53* gene mutation ($P = 0.082$). The AI was negatively associated with good histological differentiation ($P = 0.0006$), the serous tumour type ($P = 0.002$), and diffuse bcl-2 expression ($P = 0.025$). Strong bcl-2 expression was associated with endometrioid tumour type ($P = 0.002$). FIGO stage and p53 protein accumulation were the only parameters that influenced overall survival time. Thus, our results suggest that histological tumour type and grade are major determinants of spontaneous apoptosis in ovarian carcinomas; *p53* alterations do not adversely but rather positively affect spontaneous apoptosis by increasing growth fraction. This, in turn, suggests p53-independency of spontaneous apoptosis in ovarian carcinomas. © 2000 Cancer Research Campaign

**Keywords:** apoptosis; *p53* gene mutation; p53 protein accumulation; growth fraction; bcl-2; ovarian cancer; survival

Apoptosis is an important mechanism of tissue homeostasis. Alterations in cell survival may contribute to tumour development, influence tumour biology and its response to chemotherapy. Apoptosis may be triggered by p53-dependent or p53-independent mechanisms that share downstream biochemical pathways. P53-dependent apoptosis is stimulus specific. In several cell types wild-type p53 protein is necessary for induction of apoptosis after DNA damage (Lowe et al, 1993; Yonish-Rouach et al, 1993; Bellamy, 1996). Other cellular insults that provoke p53-dependent apoptosis are: non-physiological oncogene activation (Qin et al, 1994; Wagner et al, 1994; Wu and Levine, 1994; de Stanchina et al, 1998; Zindy et al, 1998), hypoxia, heat shock and cytokine deprivation (Bellamy, 1996; Evan and Littlewood, 1998). Thus, defects in p53-dependent apoptotic response may contribute to malignant transformation, tumour progression and tumour resistance to DNA damage-inducing therapy.

P53-mediated apoptosis involves transcriptional repression of *bcl-2*, activation of *bax* and *bcl-x*L, as well as immediate interaction with proteins – mediators of apoptosis (Miyashita et al, 1994*a*, 1994*b*; Miyashita and Reed, 1995; Amundson et al, 1998). Bcl-2 and bcl-$x_L$ proteins are inhibitors of programmed cell death, while bax belongs to promotors. It is suggested that both bcl-2 and bax proteins may participate in mitochondrial pore formation, and thus influence the execution step of apoptosis. Bcl-2 and bcl-$x_L$ anti-apoptotic proteins may be blocked by complex formation with bax (Reed, 1997).

Little is known on apoptosis regulation in vivo. *p53* gene alterations are very frequent in ovarian cancer – they have been found in 81% of serous type tumours in our own material, and are rather an early step in the development of ovarian cancer (Kupryjanczyk et al, 1993, 1995*a*; reviewed in Kupryjanczyk, 1996). One may expect that *p53* gene mutations will negatively influence p53-dependent spontaneous and induced apoptosis. It is assumed that poor response to DNA-damage in tumours bearing a *p53* gene mutation may cause genomic instability and tumour progression, as well as resistance to DNA damage-inducing therapy. However, studies on p53 status and response to cisplatin-based chemotherapy have not given so far equivocal results (Righetti et al, 1996; De Feudis et al, 1997). To our knowledge, spontaneous apoptosis has not been evaluated in relation to *p53* gene mutation neither in ovarian or other tumour samples. Also, nothing is known about possible prognostic value of spontaneous apoptosis in ovarian

*Received 12 January 1999*
*Revised 28 May 1999*
*Accepted 9 September 1999*

*Correspondence to:* J Kupryjanczyk cancer. The aim of this study was to evaluate associations between spontaneous apoptosis and *p53* gene mutation, p53 protein accumulation, growth fraction, bcl-2 expression, clinico-pathological parameters and overall survival in ovarian carcinomas.

### MATERIALS AND METHODS

### Patients population

Patients ranged in age from 17 to 79 years (mean 56.9). The study was performed on 71 tumours prior to chemotherapy: 64 ovarian, four tubal and three disseminated peritoneal carcinomas of mullerian type (i.e. serous). Tumours were staged according to the criteria of the International Federation of Gynaecologists and Obstetricians (Peterson et al, 1988; Creasman, 1989; Hirai et al, 1989). Ten tumours were in stage I, six were in stage IIC and 55 were in stage III or IV. Follow-up was available for 61 ovarian cancer patients and ranged from 3 to 96 months (mean 25); 24 patients died of the disease.

### Histopathological data

Tumours were classified according to the criteria of the World Health Organization (Russell, 1994). Fifty-one tumours were of the serous type and 20 of other types (seven endometrioid, four mucinous, four clear cell, one transitional cell, one carcinosarcoma, three undifferentiated). Tumour sections used for the apoptosis and immunohistochemical evaluations were re-examined as to the histological and nuclear grades according to the criteria given by Russell (1994) and Barber et al (1975) respectively (Table 1).

**Table 1** Tumour characteristics ($n = 71$)

| | |
|---|---|
| Apoptotic index | |
| Range | 0.024–0.182 |
| Mean (s.d.) | 0.112 (0.034) |
| Proliferation index | |
| Range | 3–90% |
| Mean (s.d.) | 54% (0.206) |
| Histological type | |
| Serous | 51 (72%) |
| Other | 20 (28%) |
| Histological differentiation | |
| Good | 4 (5.5%) |
| Moderate | 14 (19.5%) |
| Poor | 54 (75%) |
| Nuclear atypia | |
| Strong | 43 (60%) |
| Moderate | 24 (33%) |
| Minor | 5 (7%) |
| p53 protein accumulation | |
| Absent | 25 (35%) |
| Present | 46 (65%) |
| bcl-2 expression | |
| Negative, focal or trace | 42 (59%) |
| Diffuse: weak, moderate or strong | 29 (41%) |
| *p53* gene mutation | |
| Not detected | 20 (28%) |
| Exons 4–6 | 31 (44%) |
| Exons 7–8 | 20 (28%) |

### Molecular genetic analysis

Thirty-one tumours were previously characterized as to the *p53* gene mutation (Kupryjanczyk et al, 1993, 1995). The other 40 tumours were analysed by single strand conformational polymorphism (SSCP) method and direct sequencing. Briefly, exons 4–11 of the *p53* were amplified by polymerase chain reaction (PCR; with application of Perkin-Elmer PCR kit) on a programmable thermal cycler (Biometra) with denaturation at 94°C, annealing at 52–65°C (depending on the exon) and extension at 72°C, each for 30 s (primer sequences and other details available from the author). PCR products, denatured with 0.1 M sodium hydroxide, were loaded to polyacrylamide gels (1:39 *N,N′*-methylenebisacrylamide to acrylamide) (12% without glycerol and 10% with 10% glycerol). Electrophoresis was performed at 100 V for 16–24 h at room temperature (RT). Normal DNA, amplified in the same PCR reaction, denatured and non-denatured was run on each gel as a control. The bands were visualized by silver-staining method compilated from several procedures.

Sequencing of the SSCP variants was performed with the use of dideoxy chain termination method according to the procedure described in the Sequencing kit with sequenase version 2.0 (UBS, Amersham) (sequences of primers used available from the author). Radioactive labelling reaction was performed at 0°C for 5 min using $\alpha$-$^{32}$P-dATP. The reaction products were electrophoresed on 8% polyacrylamide gels (*N,N′*-methylenebisacrylamide and acrylamide ratio 1:19, 1 × TTE, 7M urea) at 80 W for 80–110 min. The gels were exposed to X-ray films for 1–7 days.

### Apoptosis analysis

Apoptotic cells were identified in paraffin-embedded material with the use of the TUNEL method with application of a commercially available kit (ApopTag Plus, Oncor, cat no. S7101). Apoptotic cells were counted at a 400× magnification with the use of a 0.49 cm$^2$ graticule containing 36 grid points. Only cells with a defined apoptotic morphology and of the total size corresponding to the size of tumour cell nuclei were taken into account. The counting was performed in four different areas most rich in apoptotic cells, at least to 200 counts per area (may correspond to 6400–8000 of tumour cells evaluated in each case). Areas adjacent to necrosis were avoided. Apoptotic index (AI) was defined as a proportion of the apoptotic cells to the number of tumour cell nuclei intersecting grid points.

### Immunohistochemical analysis

P53 protein accumulation, bcl-2 and Ki-67 expressions were detected immunohistochemically on paraffin-embedded material after heat-mediated antigen retrieval. We used PAb1801 monoclonal antibody (1:500, Genosys) for the p53 protein, anti-bcl-2 monoclonal antibody (1:80, Dako, Glostrup, Denmark) for bcl-2 protein, and MIB-1 antibody (1:50, Immunotech, Marseille, France) for Ki-67 antigen. Deparaffinized sections were boiled 2 × 5 min (for p53 and bcl-2) or 3 × 5 min (for Ki-67) in a citrate buffer (pH 6.0) at 700 W. Biotinylated goat anti-mouse IgG (1:1500, cat. no. 816), peroxidase conjugated streptavidin (1:500, cat. no. 309) (both from Immunotech, Marseille, France), and DAB were used as a detection system. Normal mouse IgG (1:25, Dako, Glostrup, Denmark) was used as a negative control.

 © 2000 Cancer Research Campaign

P53 protein accumulation was described as present or absent; percentage of p53-positive cells was estimated semiquantitatively. Bcl-2 expression was evaluated semiquantitatively and the following staining categories were created: (1) negative, trace, or focal positive; diffusely positive, either (2) weak, (3) moderate or (4) strong. MIB-1-positive cells were counted in three different foci most rich in proliferating cells to 500 counts per focus. A mean proliferation index (PI) was defined for each case.

### Statistical analysis

Number of apoptotic cells to the number of tumour cell nuclei was evaluated as a dependent variable in a weighed multiple logistic regression model (Williams, 1982). Independent variables included: histological tumour type (serous vs others), two binary variables indicating histological tumour grade (1 vs other, 2 vs other) and nuclear grade (2 vs other, 3 vs other), p53 protein accumulation (absent vs present), two binary variables indicating *p53* gene status (no mutation vs mutation, a mutation in exons 7–8 vs another status) and bcl-2 expression (negative, trace or focal vs diffuse of any intensity). This analysis has been performed twice, without and with inclusion of the proliferation index as an independent variable.

Bcl-2 expression was studied as a dependent variable with the use of the Kruskal–Wallis test (Siegel and Castellan, 1988). This analysis included histological tumour type and *p53* gene mutation as independent variables. Associations of bcl-2 expression with histological or nuclear grades and with a histological type were studied by a correlation coefficient gamma and Fisher's test respectively (Goodman and Kruskal, 1979; Mehta and Patel, 1983). For overall survival analysis we used Cox's model and log-rank test stratified according to FIGO stage. The level of significance was set at 5%.

### RESULTS

### *p53* status

*p53* gene alterations have been found in 28 of the 40 new tumours (70%); the mutational spectrum was similar to that already published for ovarian cancer, except that exon 5 mutations accounted for about 50% of the alterations detected. Table 2 shows a novel complex mutation of the *p53* gene and four rare alterations that have not been reported for ovarian cancer (other mutations will be published elsewhere).

Altogether, among 71 tumours *p53* gene mutations were present in 51 (72%); 31 had mutations in exons 4–6 (44%) and 20 in exons 7–8 (28%). Forty-two mutations were missense and nine were nonsense; 33 tumours showed loss of heterozygosity at *p53*. In 20 tumours (28%) no mutation has been detected in the sequences studied, i.e. exons 4–11. P53 protein accumulation was observed in 46 tumours (64%), and the percentage of positive cells ranged from 30–100% (mean 81%).

### Bcl-2 expression

Twenty tumours did not express bcl-2 protein, 11 showed only trace reactivity, and the staining was focal in another 11 tumours. Apparent diffuse bcl-2 expression of any intensity was observed in 29 tumours (41%); it was weak in 17, moderate in eight and very strong in four tumours. The latter group included a poorly differentiated serous carcinoma, and three cases of poorly differentiated endometrioid carcinoma. The association of strong bcl-2 expression with endometrioid tumour type was statistically significant ($P = 0.002$). Bcl-2 expression was not associated with other parameters studied, in particular with *p53* gene mutation or p53 protein accumulation.

### Apoptotic index

The AI ranged 0.024–0.182 (mean 0.112). In univariate evaluation mean values of the AI for good, moderate and poor histological differentiation were 0.06, 0.12 and 0.11 respectively; mean AI value for tumours without *p53* gene mutation was 0.10, while for tumours with *p53* gene mutation it was 0.11 (without differences in respect to missense or nonsense mutation, or heterozygosity status); mean AI value for tumours without, and with diffuse bcl-2 expression was 0.11 and 0.10 respectively. The multivariate logistic regression model for proportion of apoptotic cells retained the following variables at a 5% level of significance: good histological differentiation ($P = 0.0006$), the serous tumour type ($P = 0.002$), the proliferation index ($P = 0.023$) and diffuse bcl-2 expression ($P = 0.025$) (parameters estimates are shown in Table 3). In the group of tumours other than serous, clear cell and endometrioid carcinomas had the highest mean AI (0.16 and 0.12 respectively). In mucinous carcinomas the AI was lower (0.09)

**Table 2** A novel *p53* gene mutation (tumour 204) and mutations not previously reported for ovarian cancer

| Tumour no. | Organ | Type | FIGO | Exon | Amino acid | Alteration | Change |
|---|---|---|---|---|---|---|---|
| 262 | Ovary | Ser | III | 5 | 126 | TAC→TAA | Tyr to Stop |
| 98 | Peritoneum | Ser | III | 5 | 176?–178? | CCCCC→CCCC | |
| | | | | | | 1 bp del | Stop at 246 |
| 100 | Ovary | Ser | III | 6 | 199 | 31 bp del | |
| | | | | | | (13355–85) | Stop at 246 |
| 205 | Ovary | Ser | II | 7 | 230 | 10 bp del | |
| | | | | | | (14015–24) | Stop at 246 |
| 204 | Ovary | Endo | III | 8 | 296 | 22 bp del | |
| | | | | | | (14555–76) | |
| | | | | | 303 | (A)GC→(A)GT | Stop at 344 |

Ser, serous; Endo, endometrioid.

© 2000 Cancer Research Campaign

**Table 3** Parameters associated with the apoptotic index in ovarian carcinomas

| | Parameters estimates (standard error) | *t* | Two-sided *P*-value |
|---|---|---|---|
| Histological tumour type | | | |
| Serous vs other | –0.306 (0.095) | –3.226 | 0.002 |
| Histological grade | | | |
| 1 vs other | –0.777 (0.215) | –3.612 | 0.0006 |
| Nuclear grade | | | |
| 3 vs other | 0.317 (0.173) | 1.834 | 0.071 |
| Proliferation index | 0.462 (0.198) | 2.33 | 0.023 |
| Bcl-2 expression | | | |
| Diffusely positive vs negative, focal, trace | –0.178 (0.077) | –2.297 | 0.025 |
| *p53* gene mutation | | | |
| Present vs absent | 0.2164 (0.093) | 1.766 | 0.082 |

than in the serous type (0.10). In contrast to low histological grade, low nuclear grade was positively associated with the AI, but it was of borderline significance ($P = 0.071$). The presence of the *p53* gene mutation did positively influence the AI, but it was at the border of significance ($P = 0.082$). This association was much stronger ($P = 0.011$) in the initial analysis, without inclusion of the proliferation index into the model. P53 protein accumulation did not influence the AI.

### Survival analysis

Overall survival analysis was performed on the group of 61 ovarian cancer patients. It included the AI (≤ 0.10 vs > 0.10), clinical stage (FIGO I, II vs III, IV), histological grade (1, 2 vs 3), p53 protein accumulation (absent vs present), *p53* gene mutation (absent vs present) and the proliferation index (≤ 46.46 vs > 46.46). The AI and PI were cut off arbitrarily at the 33rd percentile. The only factor that influenced overall survival was FIGO stage (RR = 5.7; 95% confidence interval (CI) 1.27–25.59; $P = 0.023$) and p53 protein accumulation, when the group was stratified for FIGO (log-rank = 4.34, $P = 0.037$).

### DISCUSSION

Wild-type p53 may simultaneously induce growth arrest and apoptosis within the same cell (Liebermann et al, 1995). Biochemical pathways for these two processes are different: the first one involves transcriptional activation of *waf1*, the other – interactions with *bcl-2* family genes. These pathways are independent, but may communicate, and a switch of one signal to the other may occur (Bellamy, 1996; Amundson et al, 1998). P53-mediated control of cell proliferation and death involves other proteins also – to date at least 20 *p53* effector genes have been recognized, which participate in regulation of growth arrest, apoptosis or both (Amundson et al, 1998). The present and other studies on human tumours show a spectacular (opposite to the physiological one) effect of impaired p53 function on tumour proliferation (Henriksen et al, 1994; Kupryjanczyk et al, 1995*b*; Rohlke et al, 1997). The same cannot be observed in relation to apoptosis. We and others have not found an association between apoptosis and p53 protein accumulation (Diebold et al, 1996; McMenamin et al, 1997; Yamasaki et al, 1997). Our study has shown that *p53* gene mutation does not contribute to attenuation of spontaneous apoptosis in ovarian carcinomas. On the contrary, it has a positive influence on this process mainly by increasing growth fraction. Thus, our results suggest that spontaneous apoptosis in ovarian carcinomas does not depend on wild-type p53 protein. This is intriguing, because spontaneous apoptosis in the tumours studied might have also been a response to DNA damage, inappropriate oncogene activation and different environmental changes, such as hypoxia, that specifically induce p53.

Evidence is accumulating that cell proliferation and apoptosis are linked. Examples of proteins with both growth-promoting and pro-apoptotic function are c-*myc*, adenovirus E1A, and E2F-1 transcription factor (Evan and Littlewood, 1998). Other authors did report a positive association between apoptosis and growth fraction or mitotic index in ovarian carcinomas (McMenamin et al, 1997; Yamasaki et al, 1997).

Our study has shown that degree of histological differentiation and histological tumour type are among major determinants of spontaneous apoptosis in ovarian carcinomas. The apoptotic index was negatively associated with good histological differentiation, which was also observed by other authors (Diebold et al, 1996; Yamasaki et al, 1997). We have shown that this association is not dependent on low growth fraction in well differentiated tumours. In contrast to good histological differentiation, good nuclear differentiation showed a positive influence on spontaneous apoptosis, although it was at the border of significance. The AI may depend on histological tumour type, too. It was higher in carcinomas other than serous. In fact, among that group of tumours the clear cell and endometrioid carcinomas had the highest AI, while mucinous carcinomas had the lowest, even lower than the serous type. High AI in clear cell and endometrioid carcinomas may be dependent on strong bax expression in most cases of these histological types, as shown by Tai et al (1998).

It has been shown for some cell lines that p53 may repress transcription of *bcl-2*, and activate that of *bax* (Miyashita et al, 1994*a*, 1994*b*; Miyashita and Reed, 1995). We have not found an association between *p53* gene mutation or protein accumulation and bcl-2 expression. Bcl-2 expression was dependent on histological tumour type: the strongest expression was seen in endometrioid carcinomas (described also by Diebold et al, 1996), despite poor tumour differentiation and the relatively high AI. Generally tumours with diffuse bcl-2 expression had significantly lower AI, which is in accord with its anti-apoptotic function. Thus, evidence is accumulating that there are major differences among histological tumour types not only in respect to p53 function, but also apoptosis and expression of apoptosis-related genes.

Apoptosis and growth fraction did not gain a prognostic significance in our analysis. We have hypothesized that p53 protein accumulation may not have a prognostic value because of different biological significance of *p53* gene mutations. In our analysis, the presence of *p53* gene mutation did not influence overall survival time. The only parameters prognostically significant were clinical stage and p53 protein accumulation. Prognostic value of p53 protein accumulation has been a matter of controversy. In this aspect, our result supports studies by Klemi et al (1995), Herod et al (1996) and Rohlke et al (1997). Therefore, it seems that p53 protein accumulation (with or without underlying gene alterations) is a more adequate manifestation of impaired p53 protein function than gene alterations that include also nonsense errors without protein accumulation.

 *© 2000 Cancer Research Campaign*

### ACKNOWLEDGEMENTS

This study was supported by a grant from Polish State Committee for Scientific Research (KBN 1212/PO5/95/08). We thank Ms Elzbieta Stanczykowska for excellent technical assistance.

### REFERENCES

Amundson SA, Myers TG and Fornace AJ (1998) Roles for p53 in growth arrest and apoptosis: putting on the breaks after genotoxic stress. *Oncogene* **17:** 3287–3299

Barber RH, Sommers SC, Snyder R and Kwon TH (1975) Histologic and nuclear grading and stromal reactions as indices for prognosis in ovarian cancer. *Am J Obstet Gynecol* **15**: 795–804

Bellamy COC (1996) p53 and apoptosis. *Br Med Bull* **53**: 522–538

Creasman WJ (1989) Announcement, FIGO stages 1988, Revisions. *Gynecol Oncol* **35**: 125–127

de Feudis P, Debernardis D, Beccaglia P, Valenti M, Graniela Sire E, Arzani D, Stanzione S, Parodi S, D'Incalci M, Russo P and Broggini M (1997) DDP-induced cytotoxicity is not influenced by p53 in nine human ovarian cancer cell lines with different p53 status. *Br J Cancer* **76**: 474–479

de Stanchina E, McCurrach ME, Zindy F, Shieh SY, Ferbeyre G, Samuelson AV, Prives C, Roussel MF, Sherr CJ and Lowe SW (1998) E1A signaling to p53 involves the p19 (ARF) tumor suppressor. *Genes Dev* **12**: 2434–2442

Diebold J, Baretton G, Felchner M, Meier W, Dopfer K, Schmidt M and Lohrs U (1996) *bcl*-2 expression, p53 accumulation, and apoptosis in ovarian carcinomas. *Am J Clin Pathol* **105**: 341–349

Evan G and Littlewood T (1998) Apoptosis. A matter of life and cell death. *Science* **281**: 1317–1322

Goodman LA and Kruskal WH (1979) *Measures of Association for Cross Classifications*. Springer-Verlag: New York

Henriksen R, Strang P, Wilander E, Backstrom T, Tribukait B and Oberg K (1994) P53 expression in epithelial ovarian neoplasms: relationship to clinical and pathological parameters, Ki-67 expression and flow cytometry. *Gynecol Oncol* **53**: 301–306

Herod JJO, Eliopoulos AG, Warwick J, Niedobitek G, Young LS and Kerr DJ (1996) The prognostic significance of bcl-2 and p53 expression in ovarian carcinoma. *Cancer Res* **56**: 2178–2184

Hirai Y, Kaku S, Teshimi H, Shimuzu Y, Chen JT, Hamada T, Fujimoto I, Yamauchi K, Sakamoto A, Hasumi K and Masubuchi K (1989) Carcinoma of the fallopian tube. Experience with 15 cases. *Gynecol Oncol* **34**: 20–26

Klemi PJ, Pylkkanen L, Kiilholma P, Kurvinen K and Joensuu H (1995) p53 protein detected by immunohistochemistry as a prognostic factor in patients with epithelial ovarian carcinoma. *Cancer* **76**: 1201–1208

Kupryjanczyk J (1996) [*p53* gene mutations and p53 protein accumulation in ovarian cancer – a review]. *Nowotwory* **46**: 35–66

Kupryjanczyk J, Thor AD, Beauchamp R, Merritt V, Edgerton S, Bell DA and Yandell DW (1993) $P^{53}$ gene mutations and protein accumulation in human ovarian cancer. *Proc Natl Acad Sci USA* **90**: 4961–4965

Kupryjanczyk J, Bell DA, Dimeo D, Beauchamp R, Thor AD and Yandell DW (1995*a*) *p53* gene analysis of ovarian borderline tumors and stage I carcinomas. *Hum Pathol* **26**: 387–392

Kupryjanczyk J, Edgerton S, Effird J, Yandell DW and Thor AD (1995*b*) S phase fraction in gynecologic cancers. A comparison with p53 accumulation and *p53* gene mutation. *Path Res Pract* **191**: 705.

Liebermann DA, Hoffman B and Steinman RA (1995) Molecular controls of growth arrest and apoptosis: p53-dependent and independent pathways. *Oncogene* **11**: 199–210

Lowe SW, Ruley HE, Jacks T and Housman D (1993) p53-dependent apoptosis modulates the cytotoxicity of anticancer agents. *Cell* **74**: 957–967

McMenamin ME, O'Neil AJ and Gaffney EF (1997) Extent of apoptosis in ovarian serous carcinoma: relation to mitotic and proliferative indices, p53 expression, and survival. *Mol Path* **50**: 242–246

Mehta CR and Patel NR (1983) A network algorithm for the exact treatment of Fisher's Exact Test in R×C Contingency Tables. *J Am Stat Assn* **78**: 427–434.

Miyashita T and Reed JC (1995) Tumor suppressor p53 is a direct transcriptional activator of the human *bax* gene. *Cell* **80**: 293–299

Miyashita T, Harigai M, Hanada M and Reed JC (1994*a*) Identification of a p53-dependent negative response in the *bcl-2* gene. *Cancer Res* **54**: 3131–3135

Miyashita T, Krajewski S, Krajewska M, Wang HG, Lin HK, Liebermann DA, Hoffman B and Reed JC (1994*b*) Tumor suppressor p53 is a regulator of *bcl*-2 and *bax* gene expression in vitro and in vivo. *Oncogene* **9**: 1799–1805

Peterson F, Kolstad P, Ludwig H and Ulfelder H (1988) *Annual Report on the Results of Treatment in Gynecological Cancer*, Vol. 20. International Federation of Gynecology and Obstetrics: Stockholm

Qin XQ, Livingston DM, Kaelin WG Jr and Adams PD (1994) Deregulated transcription factor E2F-1 expression leads to S-phase entry and p53-mediated apoptosis. *Proc Natl Acad Sci USA* **91**: 10918–10922

Reed JC (1997) Double identity for proteins of the Bcl-2 family. *Nature* **387**: 773–776

Righetti SC, Della Torre G, Pilotti S, Menard S, Ottone F, Colnaghi MI, Pierotti MA, Lavarino C, Cornarotti M, Oriana S, Bohm S, Bresciani GL, Spatti G and Zunino F (1996) A comparative study of p53 gene mutations, protein accumulation, and response to cisplatin-based chemotherapy in advanced ovarian carcinoma. *Cancer Res* **56**: 689–693

Rohlke P, Milde-Langosch K, Weyland C, Pichlmeier U, Jonat W and Loning T (1997) p53 is a persistent and predictive marker in advanced ovarian carcinomas: multivariate analysis including comparison with Ki-67 immunoreactivity. *J Cancer Res Clin Oncol* **123**: 496–501

Russell P (1994) Surface epithelial-stromal tumors of the ovary. In: *Blausteins Pathology of the Female Genital Tract*, Kurman RJ (ed), pp. 705–782. Springer-Verlag: Berlin

Siegel S and Castellan NJ (1988) *Non-parametric Statistics for the Behavioral Sciences*, 2nd edn. McGraw UK: New York

Tai YT, Lee S, Niloff E, Weisman C, Strobel T and Cannistra SA (1998) BAX protein expression and clinical outcome in epithelial ovarian cancer. *J Clin Oncol* **16**: 2583–2590

Wagner AJ, Kokontis JM and Hay N (1994) Myc-mediated apoptosis requires wild-type p53 in a manner independent of cell cycle arrest and the ability of p53 to induce p21waf1/cip1. *Genes Dev* **8**: 2817–2830

Williams DA (1982) Extra-binomial variation in logistic linear models. *Applied Statistics* **31**: 144–148

Wu X and Levine AJ (1994) p53 and E2F-1 cooperate to mediate apoptosis. *Proc Natl Acad Sci USA* **91**: 3602–3606

Yamasaki F, Tokunaga O and Sugimori H (1997) Apoptotic index in ovarian carcinoma: correlation with clinicopathologic factors and prognosis. *Gynecol Oncol* **66**: 439–448

Yonish-Rouach E, Grunwald D, Wilder S, Kimchi A, May E, Lawrence J-J, May P and Oren M (1993) p53-mediated cell death: relationship to cell cycle control. *Mol Cell Biol* **13**: 1415–1423

Zindy F, Eischen CM, Randle DH, Kamijo T, Cleveland JL, Sherr CJ and Roussel MF (1998) Myc signaling via the ARF tumor suppressor regulates p53-dependent apoptosis and immortalization. *Genes Dev* **12**: 2424–2433

*© 2000 Cancer Research Campaign* 
